# Supplementary material for: Loss of NPC1 enhances phagocytic uptake and impairs lipid trafficking in microglia
Source: Nat Commun. 2021 Feb 24;12:1158. doi: 10.1038/s41467-021-21428-5 (PMC7904859; doi:10.1038/s41467-021-21428-5)
Supplement: Supplementary file 1 — Supplementary Information [file 41467_2021_21428_MOESM1_ESM.pdf]

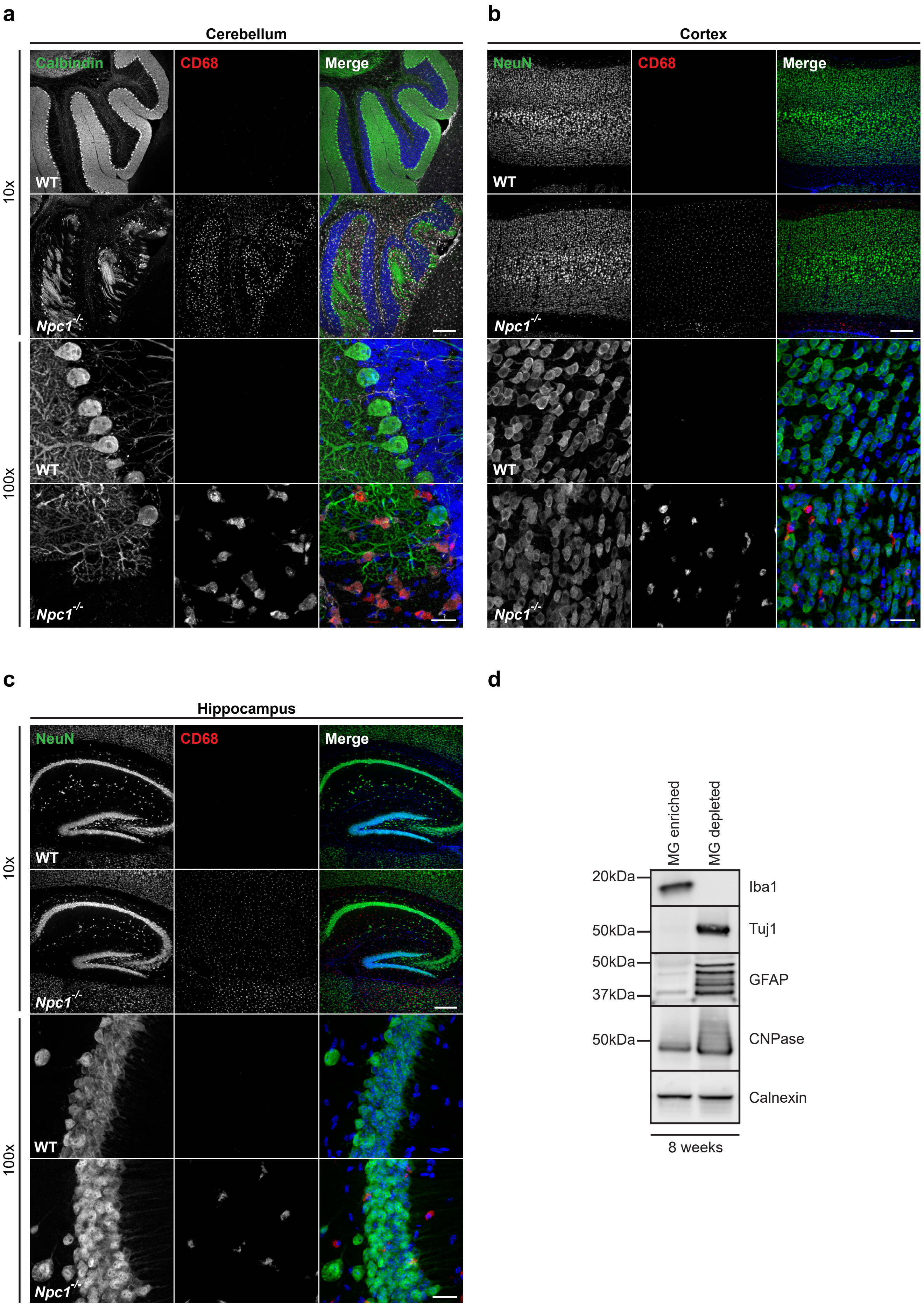


Supplementary Fig.1

**Supplementary Fig. 1. Pronounced microgliosis in symptomatic *Npc1*$^{-/-}$ mice and quality control for purity of microglial isolation. a-c** Immunostaining of cerebellum (**a**), cortex (**b**) and hippocampus (**c**) of 8 weeks old WT and *Npc1*$^{-/-}$ mice from 3 independent experiments (n=3) with antibodies against neuronal markers (green) Calbindin (**a**, Purkinje cells) and NeuN (**b-c**) and lysosomal microglial marker CD68 (red). Low magnification (10x, upper panels) analysis of *Npc1*$^{-/-}$ brain shows evident Purkinje cell loss in the cerebellum while no significant neuronal loss is observed within the cortex and hippocampus. High magnification images (100x, lower panels) show amoeboid microglial morphology in *Npc1*$^{-/-}$ brains. Hoechst was used for nuclear staining (blue). Scale bars: 250 μm (10x, upper panels) and 25 μm (100x, lower panels). **d** Quality control for purity of microglial isolation from 8 weeks old mice from 3 independent experiments (n=3) using MACS technology. Total protein lysates from microglia (MG) enriched and depleted fractions were analyzed via western blot analysis for cell specific markers of microglia (Iba1), neurons (Tuj1), astrocytes (GFAP) and oligodendrocytes (CNPase). Calnexin was used as a loading control.

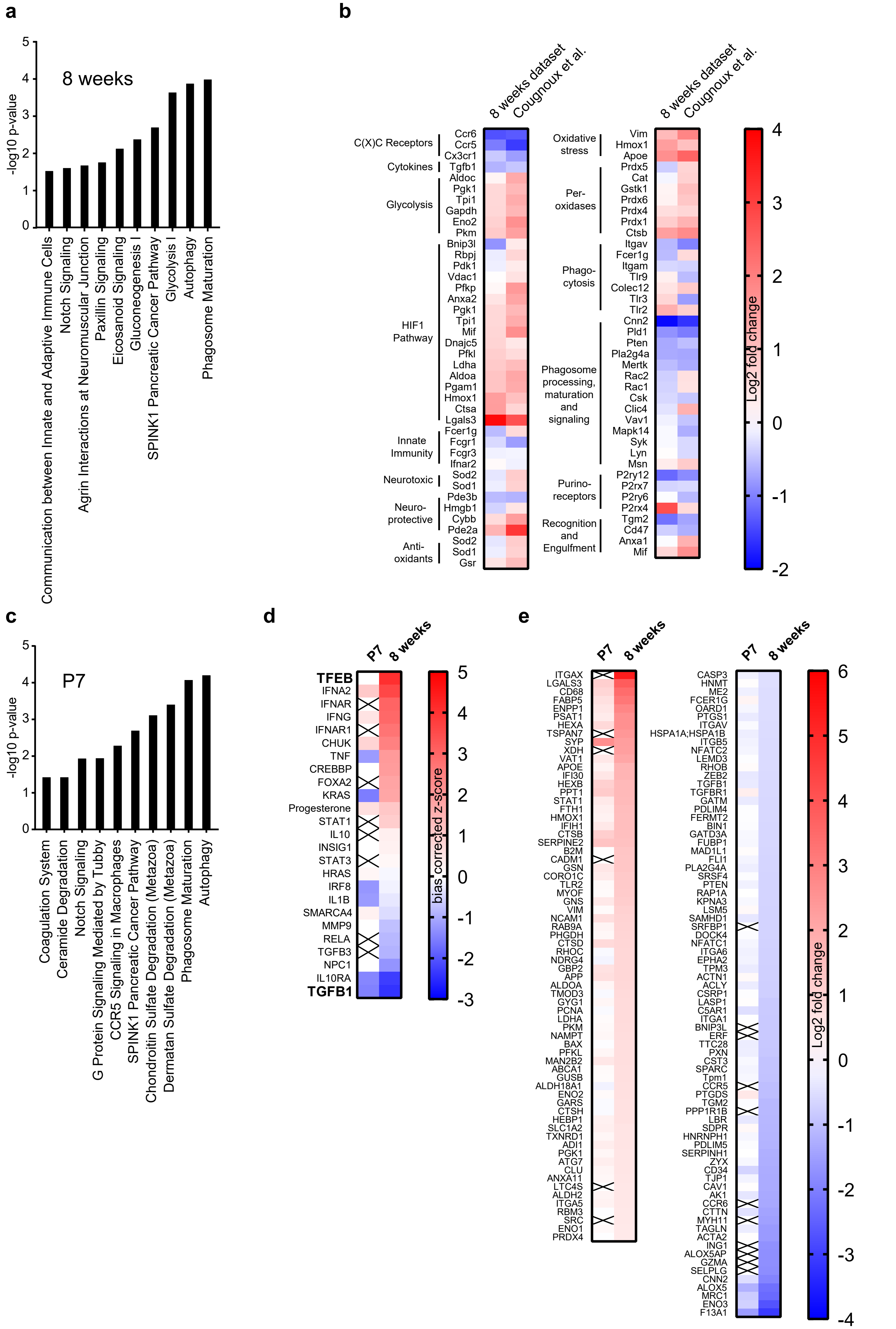


Supplementary Fig.2

**Supplementary Fig. 2. Comparative analysis of the *Npc1*$^{-/-}$ MS data. a** The ten most significantly affected canonical pathways (IPA software analysis) in microglia isolated from 8 weeks old *Npc1*$^{-/-}$ mice are represented as bar charts plotting the negative transformed log10 p-value. **b** Comparison of our proteomic dataset (8 weeks) and previously reported transcriptome study (7 weeks, Cougnoux et al.) [50] of *Npc1*$^{-/-}$ microglia. Proteomic and transcriptomic log2 fold changes of the *Npc1*$^{-/-}$ microglia are compared in a heatmap sorted according to protein functions annotated in the transcriptomic study. **c** The ten most significantly affected canonical pathways (IPA software analysis) in microglia isolated from P7 *Npc1*$^{-/-}$ mice are represented as bar charts plotting the negative transformed log10 p-value. **d** Upstream regulator analysis of MS data from P7 and 8 weeks old *Npc1*$^{-/-}$ mice using IPA. The bias corrected activation z-scores ($z > 0$: activation; $z < 0$: inhibition) are plotted in a heatmap. Crosses indicate missing values. **e** Significantly changed proteins under TGFB1 regulation in *Npc1*$^{-/-}$ microglia from P7 and 8 weeks old mice. The heatmap indicates the log2 protein fold changes. Proteins are sorted according to changes at 8 weeks. Crosses indicate missing values.

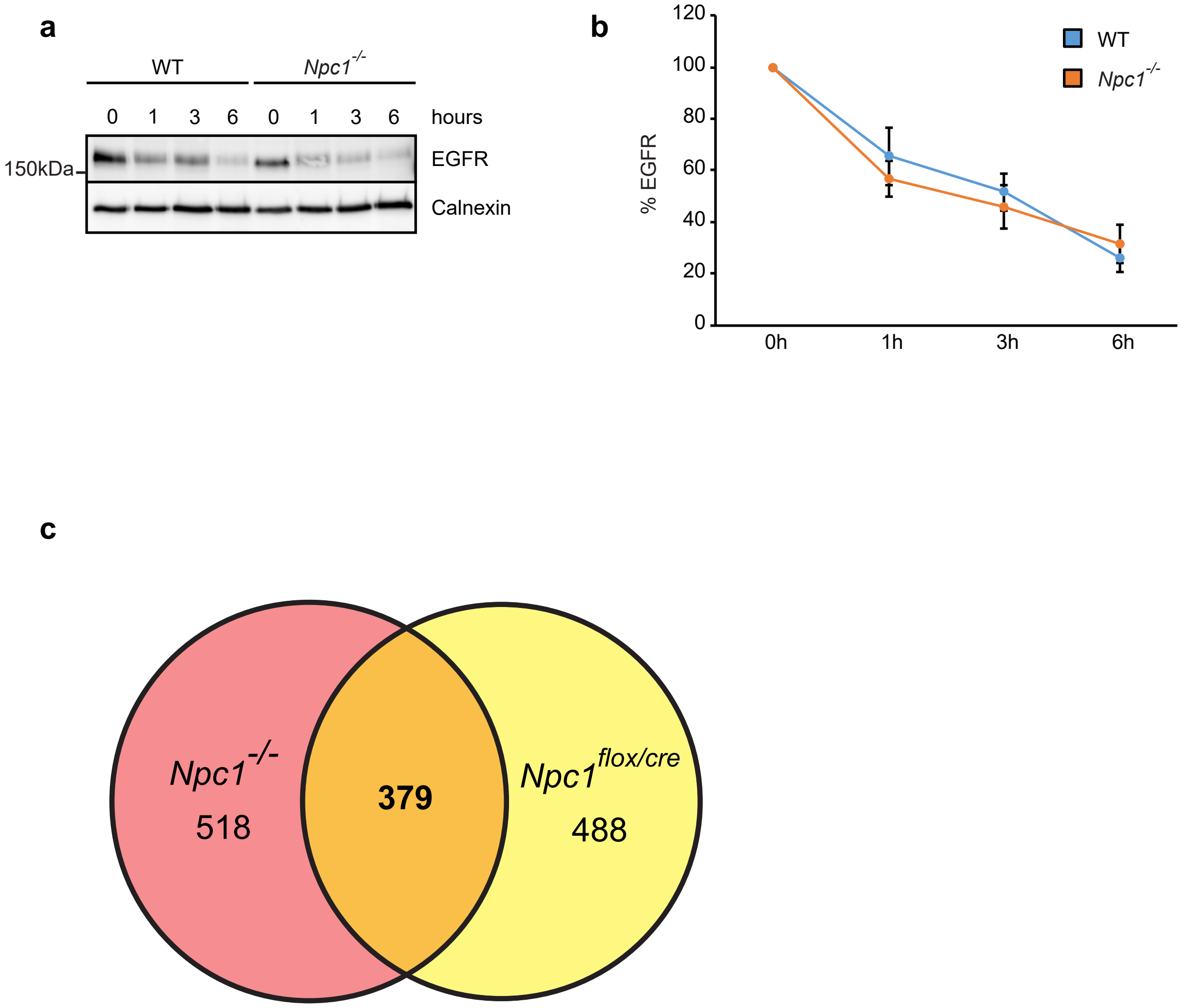


**Supplementary Fig. 3. EGFR degradation assay and comparative analysis of the *Npc1*$^{-/-}$ and *Npc1*$^{flox/cre}$ MS data. a-b** Cultured primary microglia isolated from WT and *Npc1*$^{-/-}$ mice were stimulated with recombinant murine EGF upon starving (serum free) condition to induce EGFR degradation. Cells were lysed in a time course (0-6 h) and lysates were analyzed for EGFR level via western blot. Representative immunoblot for EGFR (**a**) and corresponding quantification (**b**) showing comparable degradation rate in *Npc1*$^{-/-}$ and WT microglia. Calnexin was used as loading control. Immunoblot quantification was performed by densitometry (ImageJ – NIH) from 3 independent experiments (n = 3). Values were normalized on t = 0 for each genotype and represent mean ± SEM. **c.** Venn diagram showing significant overlap between proteomes of *Npc1*$^{-/-}$ (8 weeks) and *Npc1*$^{flox/cre}$ (5 months) microglia. Only hits with a p-value less than 0.05, FDR corrected, and with a log2 fold change larger than 0.5, or smaller than -0.5 were included into the analysis.

Supplementary Fig.3

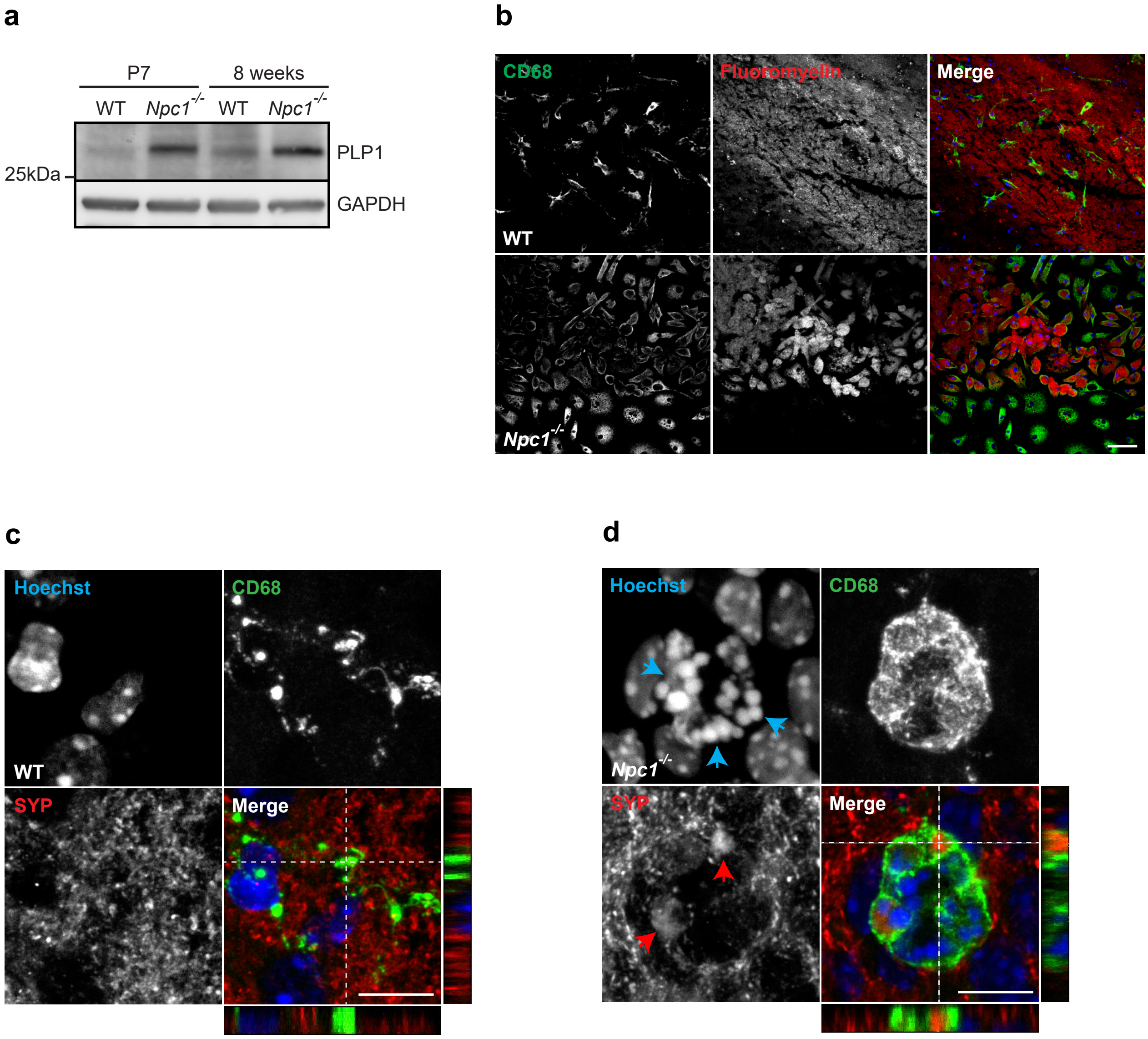


Supplementary Fig.4

**Supplementary Fig. 4. Microglia show an increased phagocytic uptake in both pre-symptomatic and symptomatic *Npc1*$^{-/-}$ mice. a** Increase of myelin protein PLP1 could be detected in acutely isolated microglia from symptomatic (8 weeks) and pre-symptomatic (P7) *Npc1*$^{-/-}$ mice from 3 independent experiments (n = 3). **b** P7 *Npc1*$^{-/-}$ microglia efficiently uptake myelin but fail in its turnover. Representative images of an ex vivo myelin phagocytic assay where acutely isolated WT and *Npc1*$^{-/-}$ microglia were plated onto an APPPS1 brain cryosection and assessed for myelin uptake in 3 independent experiments (n = 3). Microglial lysosomes were stained with an antibody against CD68 (green) while myelin was visualized using Fluoromyelin (red). Nuclei were labeled using Hoechst (blue). In contrast to intact white matter tracts visualized by Fluoromyelin upon addition of WT microglia, white matter tracts were disrupted upon addition of *Npc1*$^{-/-}$ microglia, indicating increased myelin uptake. Phagocytosed myelin accumulated within CD68 positive compartments in *Npc1*$^{-/-}$ microglia, suggesting possible impairments in myelin turnover. Scale bar: 50 µm. **c-d** Microglial phagocytosis of neuronal material in pre-symptomatic *Npc1*$^{-/-}$ mice from 3 independent experiments (n = 3). Immunostaining of WT (**c**) and *Npc1*$^{-/-}$ (**d**) brain sections using antibodies against CD68 (green) and synaptic protein SYP (red). Hoechst was used for nuclear staining (blue). WT cortical microglia (**c**) exhibits physiological CD68 punctate staining pattern (green) with no detectable nuclear or synaptic material within late endosomal/lysosomal compartments. In contrast, representative image of a single cortical *Npc1*$^{-/-}$ microglia (**d**) shows condensed nuclear signal (blue arrowheads) and SYP positive material (red arrowheads) within a CD68 positive amoeboid microglia. Scale bars: 10 µm.

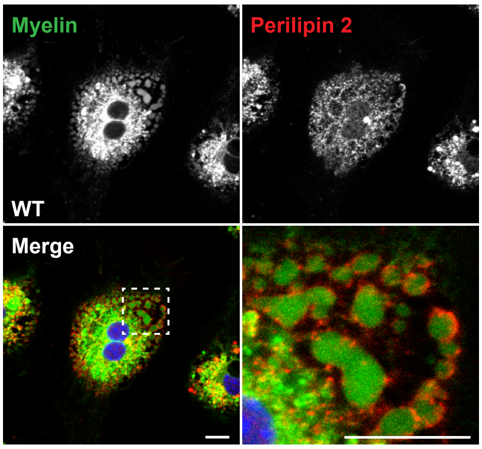


**Supplementary Fig. 5. Myelin turnover and recycling into lipid droplets.** Cultured primary microglia isolated from P7 WT mice from 3 independent experiments (n = 3) were incubated with fluorescently labeled myelin (green) and analyzed at 48 h using antibody against lipid droplet marker Perilipin 2 (red). Boxed region is enlarged in lower right panel showing that lipid vesicles forming after myelin turnover are lipid droplets. Hoechst was used for nuclear staining (blue). Scale bars: 10 µm.
