# Supplementary material for: Loss of NPC1 enhances phagocytic uptake and impairs lipid trafficking in microglia
Source: Nat Commun. 2021 Feb 24;12:1158. doi: 10.1038/s41467-021-21428-5 (PMC7904859; doi:10.1038/s41467-021-21428-5)
Supplement: Supplementary file 3 — Description of Additional Supplementary Files [file 41467_2021_21428_MOESM3_ESM.docx]

**Supplementary Data Legends**

**Supplementary Data 1. MS analysis of microglia isolated from symptomatic *Npc1^-/-^* mice.** Table displays all proteomic changes detected in the *Npc1^-/-^* versus WT mice (8 weeks), including averages of protein LFQ intensity ratios and their log2 conversion, corresponding p-values (red = p < 0.05) and log2 transformed LFQ intensities. A permutation based FDR estimation was applied and significantly changed proteins were highlighted (+). Regulated proteins are indicated with + or – for up- and down-regulation, respectively. To show the enrichment of proteomic signatures for microglial proteins, we provide color-coded relative log2 abundance (blue = decreased and red = increased) of different brain cells as reported ^1^. Microglia were analyzed from 3 independent experiments (n=3).

**Supplementary Data 2. MS analysis of microglia isolated from pre-symptomatic *Npc1^-/-^* mice.** Table displays all proteomic changes detected in the *Npc1^-/-^* versus WT mice (P7), including averages of protein LFQ intensity ratios and their log2 conversion, corresponding p-values (red = p < 0.05) and log2 transformed LFQ intensities. A permutation based FDR estimation was applied and significantly changed proteins were highlighted (+). Regulated proteins are indicated with + or – for up- and down-regulation, respectively. To show the enrichment of proteomic signatures for microglial proteins, we provide color-coded relative log2 abundance (blue = decreased and red = increased) of different brain cells as reported ^1^. Microglia were analyzed from 3 independent experiments (n=3).

**Supplementary Data 3. MS analysis of microglia isolated from 5 months old *Npc1^flox/cre-^*and *Npc1^flox/cre+^* mice.** Table displays all proteomic changes detected in the *Npc1^flox/cre+^* (*Cre+*) versus *Npc1^flox/cre-^* (*Cre-*) mice, including averages of protein LFQ intensity ratios and their log2 conversion, corresponding p-values (red = p < 0.05) and log2 transformed LFQ intensities. A permutation based FDR estimation was applied and significantly changed proteins were highlighted (+). Regulated proteins are indicated with + or – for up- and down-regulation, respectively. To show the enrichment of proteomic signatures for microglial proteins, we provide color-coded relative log2 abundance (blue = decreased and red = increased) of different brain cells as reported ^1^. Microglia were analyzed from 3 independent experiments (n=3).

**Supplementary Data 4. MS analysis of human macrophages from NPC patients.** Table displays all proteomic changes identified in peripheral blood-derived macrophages from NPC patients (NPC, n = 7) versus healthy controls (CTR, n = 3), including averages of protein LFQ intensity ratios and their log2 conversion, corresponding p-values (red = p < 0.05) and log2 transformed LFQ intensities.

**Supplementary Data 5. Demographic and clinical characteristics of NPC patients and healthy controls.** Summary of demographic and clinical data recorded for each recruited NPC patient (NPC, n = 7) and healthy control (CTR, n = 3), including age at sampling, gender, illness severity grade, identified mutation in *NPC1* gene and pharmacological treatment.

**Supplementary Data Reference**

1. Sharma, K. *et al.* Cell type- and brain region-resolved mouse brain proteome. *Nature neuroscience* **18**, 1819-1831, doi:10.1038/nn.4160 (2015).
