# Supplementary material for: Loss of NPC1 enhances phagocytic uptake and impairs lipid trafficking in microglia
Source: Nat Commun. 2021 Feb 24;12:1158. doi: 10.1038/s41467-021-21428-5 (PMC7904859; doi:10.1038/s41467-021-21428-5)
Supplement: Supplementary file 9 — Reporting summary [file 41467_2021_21428_MOESM9_ESM.pdf]

nature research

Corresponding author(s): Sabina Tahirovic

Last updated by author(s): Dec 21, 2020

Reporting Summary

Nature Research wishes to improve the reproducibility of the work that we publish. This form provides structure for consistency and transparency in reporting. For further information on Nature Research policies, see Authors & Referees and the Editorial Policy Checklist .

Statistics

For all statistical analyses, confirm that the following items are present in the figure legend, table legend, main text, or Methods section.

n/a	Confirmed	
☐	☒	The exact sample size (*n*) for each experimental group/condition, given as a discrete number and unit of measurement
☐	☒	A statement on whether measurements were taken from distinct samples or whether the same sample was measured repeatedly
☐	☒	The statistical test(s) used AND whether they are one- or two-sided<br>*Only common tests should be described solely by name; describe more complex techniques in the Methods section.*
☐	☒	A description of all covariates tested
☐	☒	A description of any assumptions or corrections, such as tests of normality and adjustment for multiple comparisons
☐	☒	A full description of the statistical parameters including central tendency (e.g. means) or other basic estimates (e.g. regression coefficient) AND variation (e.g. standard deviation) or associated estimates of uncertainty (e.g. confidence intervals)
☐	☒	For null hypothesis testing, the test statistic (e.g. *F*, *t*, *r*) with confidence intervals, effect sizes, degrees of freedom and *P* value noted<br>*Give P values as exact values whenever suitable.*
☒	☐	For Bayesian analysis, information on the choice of priors and Markov chain Monte Carlo settings
☒	☐	For hierarchical and complex designs, identification of the appropriate level for tests and full reporting of outcomes
☒	☐	Estimates of effect sizes (e.g. Cohen's *d*, Pearson's *r*), indicating how they were calculated

Our web collection on statistics for biologists contains articles on many of the points above.

Software and code

Policy information about availability of computer code

Data collection	Confocal imaging collection was done using Leica LAS X (version 3.1.5).<br>Transmission Electron Microscopy micrographs were acquired on a JEM 1400plus (JEOL) using the TEMCenter and tile scans with the ShotMeister software packages v2 (JEOL).<br>Western blot acquisition was done using ImageQuant LAS4000 (GE Healthcare, version 1.2).<br>For MS analysis, Xcalibur (version 4.1.31.9) was used for data collection.
Data analysis	Confocal imaging analysis was done using Fiji (version 1.52p).<br>MS data was analyzed with the software Maxquant (version 1.6.3.3), Perseus (version 1.6.14.0) and Spectronaut (version 12.0.20491.14.21367). The proteomic data was further analyzed through the use of Ingenuity Pathway Analysis (IPA, QIAGEN Inc., 2019-05-15).

For manuscripts utilizing custom algorithms or software that are central to the research but not yet described in published literature, software must be made available to editors/reviewers. We strongly encourage code deposition in a community repository (e.g. GitHub). See the Nature Research guidelines for submitting code & software for further information.

Data

Policy information about availability of data

All manuscripts must include a data availability statement. This statement should provide the following information, where applicable:

- Accession codes, unique identifiers, or web links for publicly available datasets
- A list of figures that have associated raw data
- A description of any restrictions on data availability

The mass spectrometry proteomics data have been deposited to the ProteomeXchange Consortium via the PRIDE partner repository with the dataset identifier PXD019447 (Npc1-/- microglia; https://www.ebi.ac.uk/pride/archive/projects/PXD019447), PXD019452 (Npc1 flox/Cre microglia; https://www.ebi.ac.uk/pride/archive/projects/PXD019452) and PXD020659 (NPC human macrophages; https://www.ebi.ac.uk/pride/archive/projects/PXD020659).

Fasta database of Mus musculus were from UniProt (https://www.uniprot.org; download: November the 5th 2018, 17005 entries).
Fasta database of Homo sapiens were from UniProt including isoforms (https://www.uniprot.org; download: December the 17th 2018, 42432 entries).
Source data are provided with this paper.

Field-specific reporting

Please select the one below that is the best fit for your research. If you are not sure, read the appropriate sections before making your selection.

- [x] Life sciences
- [ ] Behavioural & social sciences
- [ ] Ecological, evolutionary & environmental sciences

For a reference copy of the document with all sections, see nature.com/documents/nr-reporting-summary-flat.pdf

Life sciences study design

All studies must disclose on these points even when the disclosure is negative.

Sample size	Sample size has been chosen based on previous experience on microglial phenotype characterization (PMID: 32510331).
Data exclusions	No data have been excluded from the analysis.
Replication	Experimental findings have been verified in at least 3 independent experiments (exact numbers are included) and using different experimental approaches. All attempts were successfull and included in the study.
Randomization	Samples were allocated into experimental groups according to their genotype.
Blinding	Investigator was not blinded. This dod not compromise the analysis since imaging quantification was done without excluding any specimen (or part of it) using an automatic analysis software (Fiji) to exclude any possible operator influence.

Reporting for specific materials, systems and methods

We require information from authors about some types of materials, experimental systems and methods used in many studies. Here, indicate whether each material, system or method listed is relevant to your study. If you are not sure if a list item applies to your research, read the appropriate section before selecting a response.

Materials & experimental systems

n/a	Involved in the study	
[ ]	[x]	Antibodies
[x]	[ ]	Eukaryotic cell lines
[x]	[ ]	Palaeontology
[ ]	[x]	Animals and other organisms
[ ]	[x]	Human research participants
[x]	[ ]	Clinical data

Methods

n/a	Involved in the study	
[x]	[ ]	ChIP-seq
[x]	[ ]	Flow cytometry
[x]	[ ]	MRI-based neuroimaging

Antibodies

Antibodies used

Primary antibodies:
Calbindin Swant No 300 D-28k 07 (F)
Iba1 Wako 019-19741 CTJ0605
NeuN Millipore ABN78 3423204
CNPase Abcam ab6319 11-5B GR3292039-1
Synaptophysin Abcam ab32594 GR3216564-1
GFAP Dako Z-0334 20047046
Tuj1 Covance MMS-435P D13AF00117
NPC1 Abcam ab134113 EPR5209 GR286549-10
NPC2 Sigma HPA000835 B114807
LAMP1 Sigma L1418 067M4900V
Cathepsin B R&D Systems AF965 HQP0116091
Cathepsin D Novus Biologicals NBP1-50682 2G193191
mouse CD68 AbD Serotec MCA1957GA 148455
human CD68 Acris AM33123SU-N 968-1BC140310
mouse CD63 Abcam ab217345 EPR21151 GR3215608-1
human GRN Invitrogen 40-3400
ApoE Millipore AB947 L26 2984204

PLP1 Abcam ab83032
EGFR Abcam ab52894 EP38Y GR3214138-6
TGFB1 R&D Systems AF-246-NA EF0317111
human CD45 Abcam ab8216 MEM-28 GR3243739-7
Perilipin 2 Progen Biotechnik GP40 904041-01
Calnexin Stressgen ADI-SPA-860 6031925
GAPDH Abcam ab8245 GR137268-3
Abeta (home made) Yamasaki A et al. 2006 3552
GRN (home made) Gotzl JK et al. 2014 8H10
Trem2 (home made) Xiang et al. 2016 5F4
Abeta BioLegend SIG-39320 6E10
Secondary antibodies:
goat anti-rat IgG (H+L), Alexa Fluor® 488 conjugate Invitrogen A-11006
goat anti-rabbit IgG (H+L), Alexa Fluor® 488 conjugate Invitrogen A-11034
goat anti-mouse IgG (H+L), Alexa Fluor® 488 conjugate Invitrogen A-11029
goat anti-rabbit IgG (H+L), Alexa Fluor® 555 conjugate Invitrogen A-21428
goat anti-rat IgG (H+L), Alexa Fluor® 555 conjugate Invitrogen A-21434
goat anti-mouse IgG (H+L), Alexa Fluor® 555 conjugate Invitrogen A-21424
goat anti-rabbit IgG (H+L), Alexa Fluor® 647 conjugate Invitrogen A-21244
goat anti-rat IgG (H+L), Alexa Fluor® 647 conjugate Invitrogen A-21247
goat anti-guinea pig IgG (H+L), Alexa Fluor® 555 conjugate Invitrogen A-21435
goat anti-rabbit IgG (H+L), HRP Conjugate Promega W401B 0000306129
goat anti-mouse IgG (H+L), HRP Conjugate Promega W402B 0000409936
donkey Anti-Goat IgG (H+L), HRP Conjugate Dianova 705-035-003
goat anti-rat IgG antibody, HRP conjugate Merck AP136P

Validation

Antibodies used in this study are described by manufactures or specified in following publications: NPC1 (1,) NPC2 (2), LAMP1 (3), CD68 and abeta 6E10 (4), CD63 (5), GRN (6), ApoE (7), PLP1 (8), TGFB1 (9), TREM2 (10) or CTSB (6) and CTSD (11) and abeta 3552 (12).

References:

1. Cermak, S. et al. Loss of Cathepsin B and L Leads to Lysosomal Dysfunction, NPC-Like Cholesterol Sequestration and Accumulation of the Key Alzheimer's Proteins. PLoS One 11, e0167428, doi:10.1371/journal.pone.0167428 (2016).
2. Roszell, B. R. et al. Pulmonary abnormalities in animal models due to Niemann-Pick type C1 (NPC1) or C2 (NPC2) disease. PLoS One 8, e67084, doi:10.1371/journal.pone.0067084 (2013).
3. Buschow, S. I. et al. Unraveling the human dendritic cell phagosome proteome by organellar enrichment ranking. J Proteomics 75, 1547-1562, doi:10.1016/j.jprot.2011.11.024 (2012).
4. Daria, A. et al. Young microglia restore amyloid plaque clearance of aged microglia. EMBO J 36, 583-603, doi:10.15252/embj.201694591 (2017).
5. Song, L. et al. KIBRA controls exosome secretion via inhibiting the proteasomal degradation of Rab27a. Nat Commun 10, 1639, doi:10.1038/s41467-019-09720-x (2019).
6. Gotzl, J. K. et al. Common pathobiochemical hallmarks of progranulin-associated frontotemporal lobar degeneration and neuronal ceroid lipofuscinosis. Acta Neuropathol 127, 845-860, doi:10.1007/s00401-014-1262-6 (2014).
7. Monasor, L. S. et al. Fibrillar Aβ triggers microglial proteome alterations and dysfunction in Alzheimer mouse models. eLife 9, doi:10.1101/861146 (2020).
8. Wu, Y. et al. Blastomere biopsy influences epigenetic reprogramming during early embryo development, which impacts neural development and function in resulting mice. Cell Mol Life Sci 71, 1761-1774, doi:10.1007/s00018-013-1466-2 (2014).
9. Beaufort, N. et al. Cerebral small vessel disease-related protease HtrA1 processes latent TGF-beta binding protein 1 and facilitates TGF-beta signaling. Proceedings of the National Academy of Sciences of the United States of America 111, 16496-16501, doi:10.1073/pnas.1418087111 (2014).
10. Xiang, X. et al. TREM2 deficiency reduces the efficacy of immunotherapeutic amyloid clearance. EMBO Mol Med 8, 992-1004, doi:10.15252/emmm.201606370 (2016).
11. Gotzl, J. K. et al. Early lysosomal maturation deficits in microglia triggers enhanced lysosomal activity in other brain cells of progranulin knockout mice. Mol Neurodegener 13, 48, doi:10.1186/s13024-018-0281-5 (2018).
12. Yamasaki A, et al. The GxGD motif of presenilin contributes to catalytic function and substrate identification of gamma-secretase. The Journal of neuroscience : the official journal of the Society for Neuroscience 26, 3821-3828 (2006).

Animals and other organisms

Policy information about studies involving animals; ARRIVE guidelines recommended for reporting animal research

Laboratory animals

Animals were group housed under specific pathogen-free conditions. Mice had access to water and standard mouse chow (Ssniff Ms-H, Ssniff Spezialdiäten GmbH, Soest, Germany) ad libitum and were kept in a 12/12-h light–dark cycle in IVC System at the temperature of 20-22°C and humidity of 45-46%. Male and female C57BL/6J (Jackson Laboratory stock N° 000664), BALB/cNctr-Npc1m1N/J (Jackson Laboratory stock N° 003092), APPPS1 mice (Radde et al., 2006) and Npc1 flox/Cre mice (generated by crossing C57BL/6-Npc1tm1.2Apl (Elrick et al., 2010) and B6.Cx3cr1tm1.1(cre)Jung/N (Yona et al., 2013) mice) were used in this study at the age of P7/8weeks/5 months and 6 months.

Wild animals	This study does not involve wild animals.
Field-collected samples	Study did not involve field-collected samples.
Ethics oversight	All experimental procedures were performed in accordance with the German animal welfare law and approval for this work has been issued to DZNE by the government of Upper Bavaria (license number ROB-55.2-2532.Vet_02-17-075).

Note that full information on the approval of the study protocol must also be provided in the manuscript.

Human research participants

Policy information about studies involving human research participants

Population characteristics	7 clinically affected Npc1 mutation carriers and 3 healthy donors were included into this study. All characteristics including age, NPC1 mutation, severity grade and pharmacological therapy are described as follows:<br>Controls (M male, F female)<br>CTR 1: 43 years, M<br>CTR 2: 25, years, F<br>CTR 3: 31 years, F<br>NPC1 patients (M male, F female)<br>Patient 1: 18 years, M, severely ill, wheel chair bound, homozygous for c.2974G>T (p.Gly992Trp), under Miglustat therapy<br>Patient 2: 25 years, M, severely ill, wheel chair bound homozygous for c.2974G>T (p.Gly992Trp), Miglustat therapy was discontinued in 2017<br>Patient 3: 20 years, M, severely ill (palliative care), final stage, early-infantile, epilepsy homozygous for c.2861C>T (p.Ser954Leu), under Miglustat therapy<br>Patient 4: 12 years, F, severely ill (SARA Score Visit 1: 14/40), c.2130+2T>C (NM_000271) and c.3019C>G (p.Pro1007Ala), under Miglustat therapy<br>Patient 5: 32 years, M, moderately ill (SARA Score Visit 1: 8/40) c.709C>T (p.Pro237Ser) and unknown mutation, under Miglustat therapy<br>Patient 6: 34 years, M, markedly ill c.2861C>T (p.Ser954Leu) and c.3019C>G (p.Pro1007Ala), under Miglustat therapy<br>Patient 7: 36 years, M, moderately ill (SARA Score Visit 1: 9/40) homozygous for c.3182T>C (p.Ile1061Thr), under Miglustat therapy
Recruitment	Patients have been registred at the Department of Neurology, Ludwig-Maximilians University (LMU, Munich, Germany) and recruited by T.B.E., S.A.S. and M.Str. LMU is a leading tertiary referral center. There are clinics for rare disorders with specialized interdisclinary teams. This includes clinics for rare lysosomal storage diseases such as NPC where patients are recruited across the country, in cllaboration with leading patient organizations. For this study patients were recuited without bias, based on avilability and willingness to participate.
Ethics oversight	All studies were performed in accordance to the 1964 Declaration of Helsinki and were approved by the local Ethics Committee of the University of Munich. All participants gave written informed consent prior to their inclusion in the study.

Note that full information on the approval of the study protocol must also be provided in the manuscript.
